# The March of Diphtheria in the Southwest of France

**Published:** 1897-01

**Authors:** 


### THE MARCH OF DIPHTHERIA IN THE SOUTH-WEST OF FRANCE.

(Proceedings of Biological Society of Paris, November 21st, 1896.)

### Sur la marche de la diphtérie dans le sud-ouest de la France.

M. Guiraud.—L'étude des courbes de mortalité et de morbidité des affections contagieuses révèle une série de faits utiles à connaître.

Cette étude nous montre, entre autres, que ces courbes se renouvellent, que tous les quinze ou vingt ans on voit se reproduire les mêmes caractères de gravité ou de bénignite de la maladie contagieuse.

C'est ainsi, par exemple, que dans le sud-ouest de la France, la diphtérie, depuis 1892, a atteint un minimum inconnu depuis longtemps, mais qui est celui que l'on a enregistré vers 1880.

Comment ne pas compter avec ces évolutions dans l'appréciation des procédés de thérapeutique, comme avec les soins accessoires, antiseptiques ou autres, naguère inusités, comme avec les méthodes de diagnostic qui font rentrer aujourd'hui dans le cadre de la diphtérie tous les cas légers jadis laissés en dehors? —*La Semaine Médicale*, Nov. 25th, 1896.

—

### Translation.

The march of diphtheria in the southwest of France.

Dr. M. Guiraud.—The study of the variations in the mortality and virulence of contagious diseases reveals a series of facts worthy of attention.

Such study shows us, among other things, that these waves or variations repeat themselves, that every fifteen to twenty years we see repeated the same benign or grave character of the contagion. Thus, in the southwest of France, diphtheria since 1892 shows a minimum of virulence and mortality unknown for a long time, but which is the same minimum as that recorded toward 1880.

How are we to value these evolutions in our appreciations of the present therapeutic procedures, accessory, antiseptic, etc., formerly unknown?

How are we to regard the present diagnostic methods, those which cause to be entered under the name of diphtheria all light cases formerly left out?

